# Approach to Optimizing Tranexamic Acid Use in Trauma: Potential Utilization of Trauma Phenotypes

**DOI:** 10.1055/a-2806-3484

**Published:** 2026-02-16

**Authors:** Jotaro Tachino, Shunichiro Nakao, Hisatake Matsumoto, Yusuke Katayama, Tetsuhisa Kitamura, Shigeto Seno, Jun Oda

**Affiliations:** 1 Department of Traumatology and Acute Critical Medicine, Graduate School of Medicine, The University of Osaka, Suita City, Osaka, Japan; 2 Division of Environmental Medicine and Population Sciences, Department of Social and Environmental Medicine, Graduate School of Medicine, The University of Osaka, Suita City, Osaka, Japan; 3 Department of Bioinformatic Engineering, Graduate School of Information Science and Technology, The University of Osaka, Suita City, Osaka, Japan

**Keywords:** tranexamic acid, trauma phenotypes, machine learning, precision medicine

## Abstract

**Background:**

Tranexamic acid (TXA) reduces mortality in patients with trauma; however, optimal patient selection remains unclear. This study aimed to identify trauma subgroups most likely to benefit from TXA administration by integrating systematic evidence mapping with trauma phenotype analysis derived from the Japan Trauma Data Bank.

**Methods:**

We conducted a two-phase study: first, a systematic search of MEDLINE, Web of Science, and the Cochrane Library databases (inception to June 28, 2024) and identified randomized controlled trials (RCTs) evaluating TXA in trauma; second, we assessed TXA's association with mortality across phenotypes derived through machine learning–based clustering using 14 variables available during initial trauma care. Among eligible studies, control group mortality and number needed to treat (NNT) were calculated and visualized via bubble plots (size = sample size).

**Results:**

Five RCTs ( *n* = 894–20,127; published 2010–2023) and one phenotype study ( *n* = 24,058; four phenotypes) were included, all reporting mortality as an outcome. At approximately 1 month post-injury, control group mortality in RCTs ranged from 10 to 21.8%, whereas in-hospital mortality across phenotypes ranged from 3.9 to 51.4%. NNT varied from 22 to 68 (RCTs) and from 10 to 98 (phenotype study), with an inverse relationship between baseline mortality and NNT, indicating greater TXA benefit in higher-risk groups.

**Conclusion:**

This study suggests that TXA is more effective in trauma subgroups with higher baseline mortality. Phenotype-driven stratification using initial clinical data may support more targeted TXA administration and improve patient outcomes. Further research is needed to validate these phenotypes for clinical implementation.

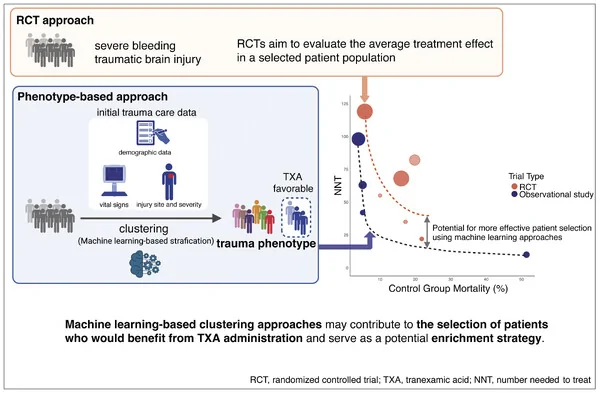

## Background

Trauma is a leading cause of mortality worldwide, particularly among younger individuals, 1 with hemorrhage and traumatic brain injury (TBI) being responsible for most acute-phase fatalities. 2 Tranexamic acid (TXA), a synthetic anti-fibrinolytic agent, functions by inhibiting plasminogen activation and fibrinolysis, thereby reducing bleeding-related mortality. 3 Although randomized controlled trials (RCTs) have demonstrated the efficacy of TXA in cases of severe trauma, its benefits in certain subgroups, such as polytrauma without major hemorrhage, minor trauma, and pediatric populations, remain unclear. 4 5 6 7 8 This uncertainty suggests that current eligibility criteria may not fully capture all patients who could benefit from TXA administration.

A 2024 machine learning study 9 analyzed patients with blunt trauma, including those typically excluded from RCTs, and identified potential subgroups that may benefit from TXA. These findings highlight the potential of machine learning–based clustering in identifying previously unrecognized patient subgroups who might benefit from TXA administration. Such an approach may inform enrichment strategies 10 11 and enhance clinical decision-making beyond the traditional clinical criteria.

This study aimed to identify patients with trauma who may benefit from TXA administration through a two-phase approach: (1) a systematic analysis of RCT data to quantify the effects of TXA across heterogeneous trauma populations, and (2) a trauma phenotype analysis using the Japan Trauma Data Bank to detect high-benefit subgroups that may be overlooked by conventional criteria.

## Methods

### Ethics Approval and Consent to Participate

This study was approved by the Ethics Committee of the University of Osaka (IRB approval no. 16260–4). The requirement for informed consent was waived owing to the retrospective analysis of anonymized data from the Japan Trauma Data Bank, in accordance with the ethical guidelines for medical research involving human subjects issued by the Ministry of Health, Labor, and Welfare of Japan.

### Study Design

We conducted a secondary analysis on the use of TXA using a two-phase methodological approach for data collection:

- First phase: A systematic analysis of RCTs evaluating the efficacy of TXA in patients with trauma.
- Second phase: An examination of trauma phenotype data from a retrospective cohort study to identify patient subgroups who may benefit from TXA administration.

We adopted this integrated approach, combining data from RCTs or observational studies, to address key limitations inherent in each study type. RCTs provide high internal validity but often lack external generalizability due to strict inclusion criteria. Conversely, observational studies, such as trauma phenotype analyses, can reveal real-world heterogeneity and identify patient subgroups based on patterns of clinical characteristics rather than isolated variables.

To address methodological challenges in comparing different study designs, we applied standardized outcome measures and focused on the association between baseline mortality risk in control groups and the observed treatment effect across the mortality risk spectrum. The primary outcome was mortality at 28 to 30 days post-injury. Secondary outcomes, when available, included bleeding- and TBI-related mortality.

### Eligibility Criteria (First Phase)

We included original RCTs that evaluated the effectiveness of TXA in patients with trauma. Studies were excluded if they met any of the following criteria: non-RCT design; sample size <300 participants (due to limited statistical power to detect clinically meaningful differences 12 and potential to increase heterogeneity in meta-analyses 13 ); duplicate publications or secondary analyses; absence of mortality-related outcomes; use of non-standard TXA administration protocols; non-English publications.

### Literature Search and Study Selection (First Phase)

We conducted a comprehensive literature search in MEDLINE, Web of Science, and the Cochrane Library from database inception through June 28, 2024, to identify the RCTs evaluating TXA efficacy in patients with trauma. The complete search strategies for each database are provided in the Supplementary Material (available in the online version only).

Two independent reviewers (J.T. and S.N.) screened the article titles and abstracts using Covidence software (Covidence, Melbourne, Australia). Disagreements were resolved through discussion with a third reviewer (Y.K.). Articles that met the inclusion criteria and those deemed potentially eligible or unclear underwent full-text review. The reasons for exclusion were documented when applicable.

The following data were extracted from each study: study design, target population, sample size, and reported primary and secondary outcomes. For studies reporting mortality as an outcome, we specifically recorded the control group mortality rate, TXA group mortality rate, and available effect measures, including relative risk and odds ratio, with their corresponding 95% confidence intervals.

### Trauma Phenotype Study (Second Phase)

Trauma phenotype classification was developed through machine learning clustering analysis using 14 clinical variables available during the initial trauma assessment. 14 These variables included patient demographics, vital signs, and Abbreviated Injury Scale (AIS) scores across six body regions. In the study, these trauma phenotypes were derived from the Japan Trauma Data Bank dataset ( *n* = 42,780; January 2013 to June 2015) with subsequent validation of phenotype reproducibility using an independent cohort ( *n* = 28,258; July 2015 to December 2017).

The clustering algorithm used silhouette analysis of standardized data using Euclidean distance, with the optimal clusters (eight distinct phenotypes) determined through average silhouette scoring and k-means methodology. Each phenotype demonstrated characteristic patterns of injury severity and anatomical distribution. Notably, one high-mortality phenotype was associated with dysregulated inflammatory and coagulation pathways, as confirmed through proteomic analysis.

Building on this foundation, a subsequent study analyzed data from the Japan Trauma Data Bank from 53,703 trauma cases (2019–2021) to evaluate the association between TXA administration and survival outcomes across phenotypes. 9 Among the eight phenotypes, four phenotypes (1, 2, 6, and 8) demonstrated particularly favorable survival outcomes with TXA administration:

- Trauma phenotype 1: Thoracoabdominal trauma with significant hemorrhage (30% transfusion rate).
- Trauma phenotype 2: Mild head injury and thoracic and extremity injuries with stable vital signs.
- Trauma phenotype 6: Head and thoracic trauma with moderate transfusion requirements (20% of cases).
- Trauma phenotype 8: Severe head or thoracic trauma or both with hemodynamic instability and high mortality rate.

In the present study, we focused specifically on these four TXA-responsive trauma phenotypes, which were associated with improved survival outcomes.

### Japan Trauma Data Bank

The Japan Trauma Data Bank is a nationwide trauma registry established in 2003, collecting data from 303 tertiary care and emergency centers across Japan. The registry primarily includes patients with suspected injuries having an AIS score ≥3, tracking their clinical course from admission to hospital discharge or death. The Japan Advanced Trauma Evaluation and Care guidelines 15 highlight the importance of administering TXA within 3 hours of injury in patients at a high risk of bleeding or with mild to moderate TBI, as an adjunctive hemostatic therapy, based on evidence from two large RCTs (CRASH-2 and CRASH-3). 4 6 Medical institutions generally adhere to these guidelines; however, the degree of adherence varies across facilities.

### Analysis

To evaluate and compare the effectiveness of TXA across studies, we used the number needed to treat (NNT) as the primary standardized metric. This clinically intuitive measure, derived from absolute risk differences between intervention and control groups, facilitates meaningful comparisons across heterogeneous trauma populations and healthcare settings. 16 For each included study, we examined control group mortality rates, TXA group mortality rates, and NNT values calculated from absolute numbers and their interquartile range derived from risk ratios, based on the reported number of analyzed participants and actual event occurrences.

Adjusted mortality rates for each phenotype were calculated using the data from the trauma phenotype study. 9 Following multiple imputation for missing values, we applied inverse probability of treatment weighting to account for potential confounding variables, including age, sex, comorbidity count, vital signs at admission, and trauma severity. These adjustments enabled the calculation of NNT values from previously reported adjusted odds ratios. 17 18

For data visualization, we generated a plot incorporating control group mortality, NNT estimates, and study sample sizes. All statistical analyses were performed using R version 4.3.2 (R Foundation for Statistical Computing, Vienna, Austria; https://www.r-project.org/ ).

## Results

**Fig. 1:**
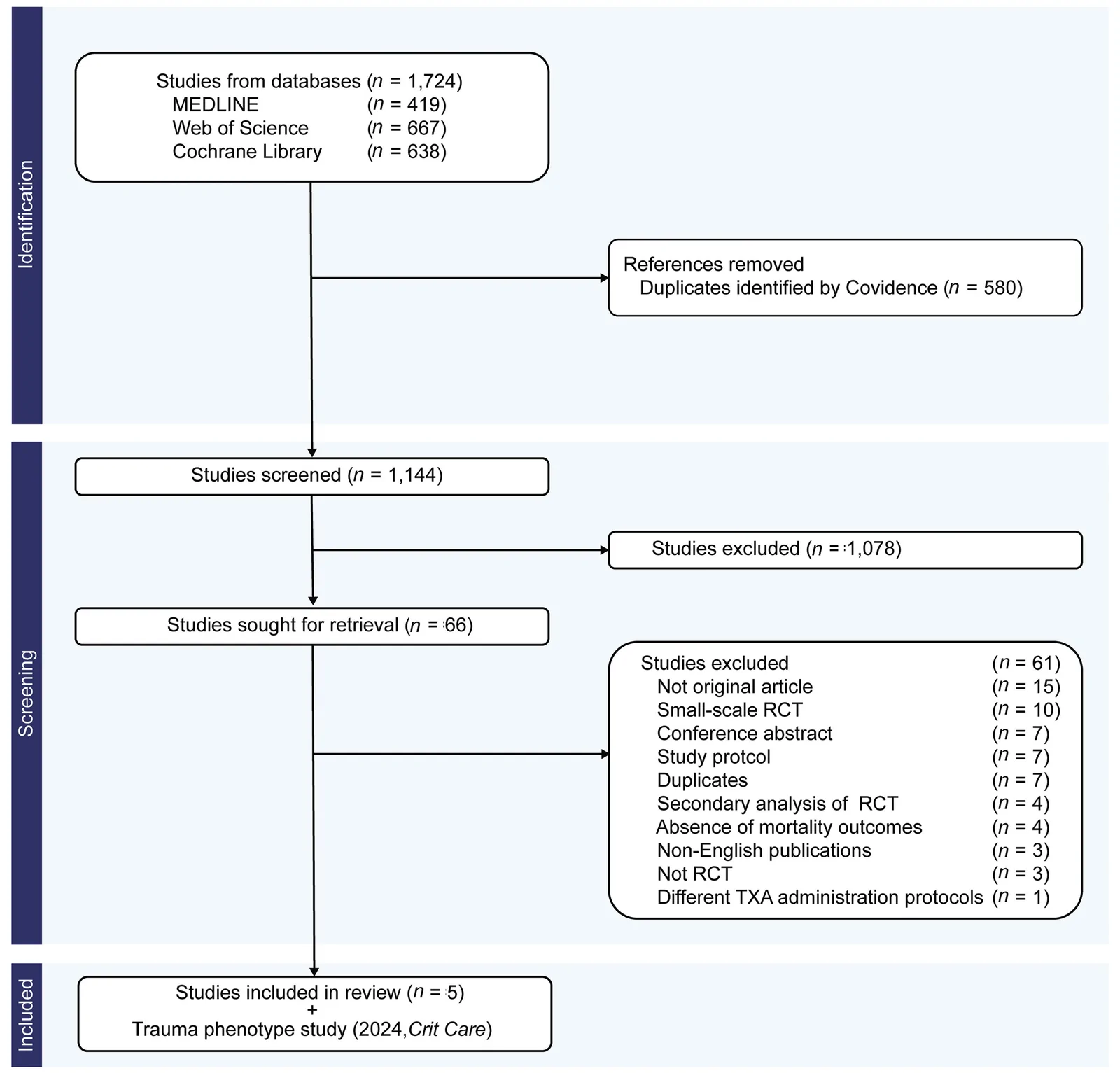
Flowchart of the selection process. RCT, randomized controlled trial; TXA, tranexamic acid.

The database search yielded 1,144 potentially relevant articles in the initial screening phase, of which 66 studies underwent full-text review to assess eligibility. A total of 61 studies were excluded for the following reasons: 15 were not original research articles, 10 were small-scale RCTs (<300 participants), 7 were conference abstracts, 7 were study protocols, 7 were duplicate publications, and 15 were excluded for other specified reasons. Ultimately, five studies met all the predetermined inclusion criteria and were selected for analysis ( Fig. 1 ). 4 6 7 8 19

In the second phase of our investigation, we included an additional study examining the association between TXA and outcomes across trauma phenotypes, 9 bringing the total number of included studies to six.

### Characteristics of the Included Studies

The characteristics and key data of the included studies are summarized in Table 1 . All five RCTs were double-blind, placebo-controlled trials, enrolling 894 to 20,127 participants. The trauma phenotype study included 24,058 patients across four phenotypes.

**Table 1:** Characteristics and key data of included studies

Trial		Patient	Outcome		Number of analyzed patients	Control group mortality (%)	TXA group mortality (%)	RR (95%CI)	NNT from raw data	NNT calculated from RR (95%CI)
2010 *Lancet* , CRASH–2 ^4^		Adult patients with trauma (≥16 years of age) with or at risk of significant bleeding	28-day all-cause of death	Primary outcome	20,127	16	14.5	0.91 (0.85–0.97)	68	69 (42–208)
			28-day bleeding death	Secondary outcome	20,127	5.7	4.9	0.85 (0.76–0.96)	119	117 (72–439)
			28-day TBI-related death	Secondary outcome	20,127	6.2	6	0.97 (0.87–1.08)	573	538 (124–infinity)
2019 *Lancet* , CRASH–3 6		Adults with TBI who were within 3 hours of injury; had a GCS score ≤12 or any intracranial bleeding on CT scan; and no major extracranial bleeding	28-day TBI-related death	Primary outcome	9,127	19.8	18.5	0.94 (0.86–1.02)	82	84 (36–infinity)
2020 *JAMA Surg* , STAAMP 7		Adults (≥18 years of age) with prehospital hypotension (SBP ≤90 mm Hg) or tachycardia (HR ≥110/min) before arrival within an estimated 2 hours of injury	30-day all-cause of death	Primary outcome	894	10	8.1	0.82 (0.60–1.11)	55	56 (36–infinity)
2020 *JAMA* 19		Out-of-hospital patients with TBI (≥15 years of age) with GCS score ≤ 12 and SBP ≥ 90 mm Hg	28-day all-cause of death	Secondary outcome	966	17.2	14.3	0.83 (0.61–1.14)	35	34 (15–infinity)
2023 *NEJM* , PATCH 8		Adults (≥18 years of age) with major trauma who were at risk for trauma-induced coagulopathy	28-day all-cause of death	Secondary outcome	1,290	21.8	17.3	0.79 (0.63–0.99)	22	12 (36–459)
2024 *Crit Care* 9	TP–1	All patients with blunt trauma registered with the JTDB	In-hospital death	Primary outcome	4,791	7.0	4.6	NA	NA	63
	TP–2				14,579	5.8	4.3	NA	NA	98
	TP–6				1,920	7.7	4.7	NA	NA	42
	TP–8				2,768	53.4	42.2	NA	NA	10

Abbreviations: CI, confidence interval; GCS, Glasgow Coma Scale; HR, heart rate; JTDB, Japan Trauma Data Bank; NNT, number needed to treat; RR, relative risk; SBP, systolic blood pressure; TBI, traumatic brain injury; TP, trauma phenotype; TXA, tranexamic acid.

A standardized TXA dosing regimen emerged as the most common protocol, consisting of a 1-g loading dose administered over 10 minutes followed by a maintenance infusion of 1 g over 8 hours. However, treatment timing and settings varied. CRASH-2 implemented treatment within 8 hours of injury, 4 whereas CRASH-3 restricted administration to a 3-hour window. 6 Three trials (STAAMP, 7 PATCH, 8 and the 2020 JAMA study 19 ) initiated TXA in prehospital settings. Both STAAMP and the 2020 JAMA incorporated adaptive protocols based on clinical reassessments or alternative dosing regimens. 7 19

Regarding primary outcomes, two trials (33%) assessed in-hospital mortality within 28 or 30 days of injury, 4 7 whereas two others (33%) assessed neurological outcomes using the Glasgow Outcome Scale-Extended, with scores >4 indicating moderate disability or good recovery at 6-month follow-up. 8 19 Of the remaining studies, one (17%) evaluated TBI-related in-hospital mortality within 28 days 6 and one (17%) evaluated all-cause in-hospital mortality.

### Distribution of Control Group Mortality and NNT

In the CRASH-2 trial, 4 analyses of all-cause mortality showed a moderate control group mortality rate (16.0%) with an NNT of 68. Bleeding-specific mortality analysis showed a lower control group mortality (5.7%) with a higher NNT of 119. The PATCH trial 8 exhibited the highest control group mortality among RCTs (21.8%) with the most favorable NNT of 22.

**Fig. 2:**
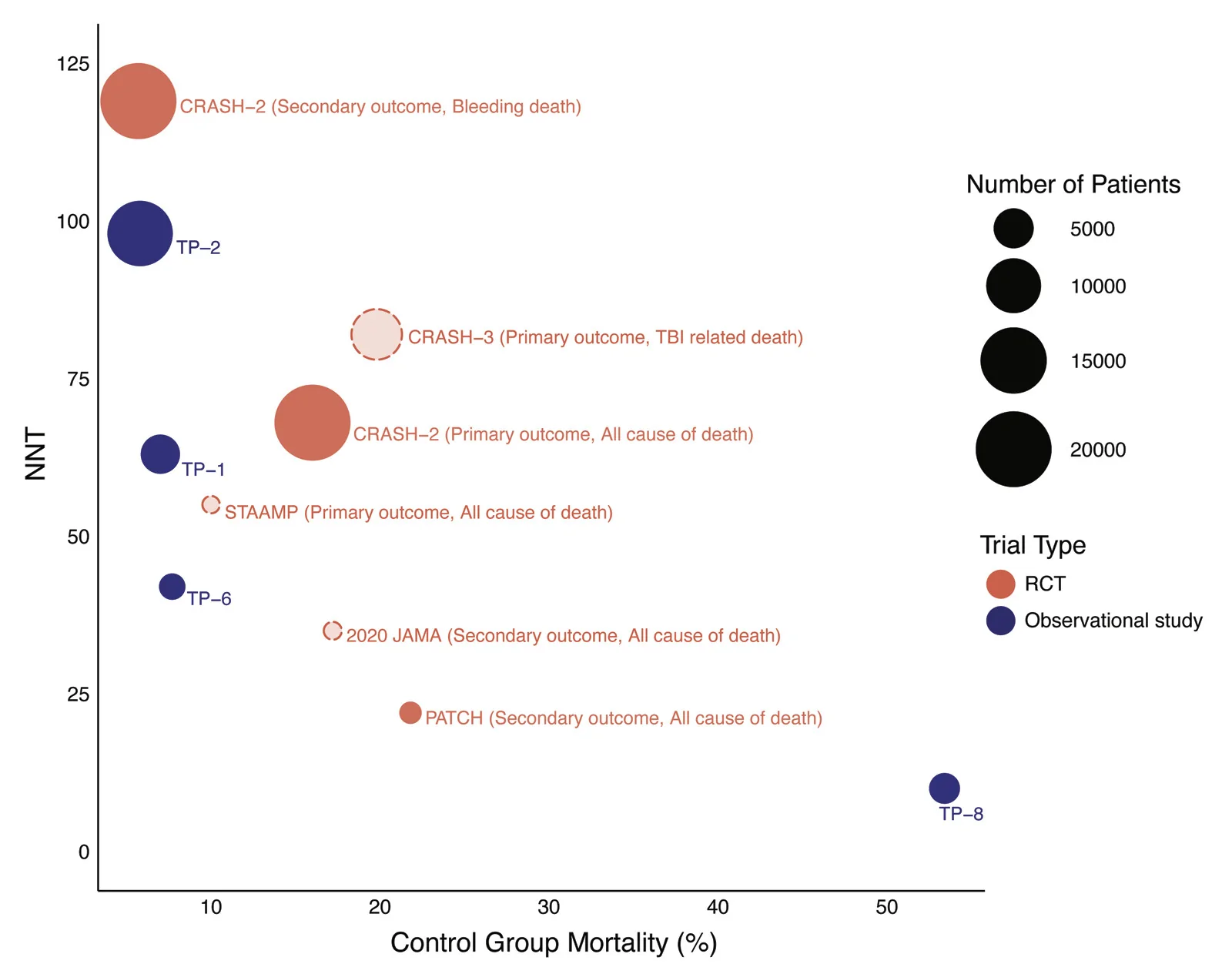
Relationship between control group mortality and number needed to treat (NNT) across included studies. The bubble size reflects the study sample size, with darker shading indicating statistically significant outcomes. Parenthetical notations specify outcome measures: bleeding (bleeding-related mortality), traumatic brain injury (TBI) (TBI-related mortality), or unspecified (28-/30-day all-cause mortality). The shading in each plot indicates statistical significance, with darker plots representing studies with statistically significant results. RCT, randomized controlled trial; TP, trauma phenotype.

The trauma phenotype analysis revealed even greater variation, with control group mortality ranging from 5.8% (trauma phenotype 2) to 53.4% (trauma phenotype 8). This mortality spectrum was inversely correlated with NNT values (98 and 10, respectively). Fig. 2 illustrates this relationship across all included studies, with circle sizes proportional to sample sizes. The consistent inverse relationship between control group mortality and NNT observed across both traditional RCTs and phenotype-based analysis suggests that the therapeutic benefit of TXA increases with baseline mortality risk.

## Discussion

In this study, we conducted a secondary analysis and used a dual methodological approach for data collection to identify the potential target populations for TXA administration in trauma care. Our integrated analysis demonstrated that higher control group mortality was associated with greater improvements in survival outcomes with TXA administration, as reflected by lower NNTs in higher-risk populations. Traditional TXA administration criteria have relied on broad, singular metrics, such as vital signs or anatomical injury sites. However, the trauma phenotype approach identified more specific, responsive subgroups beyond these traditional classifications. These findings support a precision application of TXA in trauma care.

This study revealed two principal insights. First, our findings relate to risk stratification and the known principles of treatment effect heterogeneity. Intervention benefit is often limited in “least sick” patients (due to good prognosis), rises with risk to a “sweet spot,” and may decrease again in the “most sick” patients where treatment is futile. 20 Our findings appear to align with this spectrum. The highest-risk group observed in our cohort, trauma phenotype 8 (53.4% mortality), was associated with the greatest absolute benefit (NNT 10), suggesting that it may be near this “sweet spot” of maximal effect. Furthermore, our observational data were associated with significant benefits even in the low-to-moderate risk trauma phenotypes 1, 2, and 6 (mortality 5.8–7.7%; NNT 42–98). This suggests that objective clustering may help identify subgroups (e.g., trauma phenotype 2) who may derive significant benefit but might be overlooked by conventional criteria.

Second, our study integrated insights from novel machine learning–derived trauma phenotypes and contextualized them within the overall trauma patient population from an NNT perspective. Recent meta-analyses have demonstrated that TXA administration reduces mortality in patients with trauma (odds ratio, 0.89; 95% confidence interval, 0.84–0.95) with an estimated NNT of 61 to prevent one additional death at 1 month. 21 Subgroup analyses from these studies also suggest that treatment effects vary between TBI and general trauma populations. 21 Our trauma phenotype approach extends beyond these traditional anatomical classifications. A key strength of our approach is that it initially encompasses all patients with trauma and objectively classifies them into distinct phenotypes. This enables the identification of candidates most likely to benefit from TXA.

The Japan Trauma Data Bank includes patients of all ages with trauma and anticipated AIS scores ≥3. Research examining the association between TXA administration and outcomes across trauma phenotypes using these data suggests favorable outcomes in trauma phenotypes 1, 2, 6, and 8. 9 Although RCT populations with control group mortality <10% showed NNTs exceeding 100, groups identified by trauma phenotypes demonstrated NNT values of 50 to 100, even with similar control group mortality, suggesting improved patient selection through heterogeneity reduction. Validating the therapeutic effects of TXA in potentially responsive subgroups could represent a novel precision medicine approach, enabling identification of more efficient and responsive populations.

These findings align with current guideline recommendations and extend existing knowledge by optimizing patient selection. Current European and Advanced Trauma Life Support guidelines recommend administering TXA within 3 hours of injury for patients with active bleeding or at significant risk of hemorrhage. 22 23 The trauma phenotype approach can be integrated within this therapeutic window, as phenotypes can be identified early in clinical care. This enables more targeted TXA administration for patients most likely to benefit while maintaining adherence to established protocols.

The inverse relationship between control group mortality and NNT observed in our analysis (e.g., the high benefit in trauma phenotype 8) raises the “all versus precision” debate. Although TXA is generally considered safe, and major RCTs have found no increased risk of thrombotic events, 4 6 7 8 19 a “one-size-fits-all” strategy may be suboptimal. Recent large RCTs failed to show benefit in their primary outcomes, 6 7 8 19 suggesting futility in broad populations. This lack of benefit is likely due to dilution of the treatment effect, as these trials included heterogeneous patients, a possibility already discussed. 8

Our findings suggest that objective clustering is crucial for optimization as it may identify high-benefit subgroups (such as trauma phenotypes 2 and 6) missed by traditional “high-risk” criteria, thereby overcoming the dilution effect. Therefore, this approach may refine clinical decision-making and suggest that the potential applications of TXA may extend beyond current indications.

This study has some limitations. First, the selection of target populations and reported outcomes varied across the included studies, complicating the extraction of directly comparable information. Differences in TXA administration protocols and eligibility criteria across studies might have influenced the results. Although we used NNT as a standardized metric to facilitate comparisons across these heterogeneous studies, this approach did not eliminate the underlying methodological differences. Rather, NNT provided a clinically interpretable framework that helped contextualize treatment effects despite these variations. Second, data on TXA administration based on trauma phenotypes were obtained from observational studies and did not verify TXA-mediated effects, thus limiting causal inference. As this phenotype analysis represents a derivation step, these approaches must be validated externally before use in clinical practice. Additionally, as the trauma phenotype study used data from Japanese trauma centers, the generalizability of these findings to different healthcare settings or patient populations may be limited. Therefore, prospective external validation of TXA across trauma phenotypes is essential.

Our findings suggest several possibilities for future studies. First, the prospective validation of trauma phenotypes and their interaction with TXA effectiveness in diverse trauma populations would strengthen the evidence base for phenotype-guided treatment. Second, developing and validating rapid phenotype identification tools based on these phenotypes could facilitate implementation for clinical use. Finally, mechanistic studies exploring the physiological basis for differential TXA responses across phenotypes, including the timing of administration and biological markers, may inform the development of more targeted anti-fibrinolytic approaches.

## Conclusion

Our integrated analysis identified a wide range of NNT values for TXA administration across different trauma populations. Patients with higher control group mortality showed consistently lower NNTs, suggesting greater potential benefits in these groups. Trauma phenotypes, derived from readily available clinical information, may serve as a practical enrichment strategy to improve patient selection and optimize the use of TXA in trauma care.

## Notes

**What is known about this topic?**

- Tranexamic acid (TXA) reduces mortality in trauma patients based on randomized controlled trials, but optimal patient selection criteria remain unclear.
- Current patient selection criteria for TXA administration, such as hemorrhage risk or traumatic brain injury, may not capture all patients who could benefit.
- Traditional randomized controlled trials have limited external generalizability due to strict inclusion criteria, potentially missing real-world patient subgroups.

**What does this paper add?**

- This study demonstrates an inverse relationship between baseline mortality risk and number needed to treat, showing TXA is most effective in higher-risk trauma populations.
- This study integrates systematic evidence from randomized controlled trials with machine learning–derived trauma phenotypes to objectively identify optimal TXA candidates beyond conventional criteria.
- This study provides a practical framework for precision TXA administration using readily available clinical data during initial trauma assessment, potentially improving patient selection and outcomes.
