# Supplementary material for: Approach to Optimizing Tranexamic Acid Use in Trauma: Potential Utilization of Trauma Phenotypes
Source: Thromb Haemost. 2026 Feb 16;126(10):1006–13. doi: 10.1055/a-2806-3484 (PMC13600770; doi:10.1055/a-2806-3484)
Supplement: Supplementary file 1 [file 10-1055-a-2806-3484_28151893.pdf]

Supplementary Information

Approach to optimising tranexamic acid use in trauma: Integrating evidence and trauma phenotypes

Supplementary Appendix: Search strategy

Database/platform:	MEDLINE
Data coverage:	Database inception to present
Date of search:	June 28, 2024
Search query and limits:	1. tranexamic acid.mp<br>2. TXA.mp<br>3. 1 or 2<br>4. trauma*.mp<br>5. injur*<br>6. 4 or 5<br>7. randomi?ed.mp<br>8. randomly.mp<br>9. RCT.mp<br>10. 7 or 8 or 9<br>11. 3 and 6 and 10
Number of hits:	419

Database/platform:	Web of Science
Data coverage:	Database inception to present
Date of search:	June 28, 2024
Search query and limits:	1. ALL=(tranexamic acid)<br>2. ALL=(TXA)<br>3. #1 OR #2<br>4. ALL=(trauma*)

	5. ALL=(injur*)<br>6. #4 OR #5<br>7. ALL=(randomi?ed)<br>8. ALL=(randomly)<br>9. ALL=(RCT)<br>10. #7 OR #8 OR #9<br>11. #3 AND #6 AND #10
**Number of hits:**	667

**Database/platform:**	**Cochrane Library**
**Data coverage:**	Database inception to present
**Date of search:**	June 28, 2024
**Search query and limits:**	#1. tranexamic acid<br>#2. TXA<br>#3. 1 OR 2<br>#4. trauma*<br>#5. injur*<br>#6. 4 OR 5<br>#7. randomi?ed<br>#8. randomly<br>#9. RCT<br>#10. 7 OR 8 OR 9<br>#11. 3 AND 6 AND 10
**Number of hits:**	638
